# Dynamic Fluctuation of Circulating Tumor Cells during Cancer Progression

**DOI:** 10.3390/cancers6010128

**Published:** 2014-01-15

**Authors:** Mazen A. Juratli, Mustafa Sarimollaoglu, Dmitry A. Nedosekin, Alexander V. Melerzanov, Vladimir P. Zharov, Ekaterina I. Galanzha

**Affiliations:** 1 Phillips Classic Laser and Nanomedicine Laboratories, University of Arkansas for Medical Sciences, Little Rock, AR 72205, USA; E-Mails: mjuratli@uams.edu (M.A.J.); msarimollaoglu@uams.edu (M.S.); danedosekin@uams.edu (D.A.N.); zharovvladimirp@uams.edu (V.P.Z.); 2 Arkansas Nanomedicine Center, University of Arkansas for Medical Sciences, Little Rock, AR 72205, USA; 3 Moscow Institute of Physics and Technology (MIPT), Moscow Region, 141700, Russia; E-Mail: m83071@gmail.com

**Keywords:** circulating tumor cells (CTCs), CTC dynamics, quantitative heterogeneity, *in vivo* flow cytometry, photoacoustics, personalized diagnosis and therapy

## Abstract

Circulating tumor cells (CTCs) are a promising diagnostic and prognostic biomarker for metastatic tumors. We demonstrate that CTCs’ diagnostic value might be increased through real-time monitoring of CTC dynamics. Using preclinical animal models of breast cancer and melanoma and *in vivo* flow cytometry with photoacoustic and fluorescence detection schematics, we show that CTC count does not always correlate with the primary tumor size. Individual analysis elucidated many cases where the highest level of CTCs was detected before the primary tumor starts progressing. This phenomenon could be attributed to aggressive tumors developing from cancer stem cells. Furthermore, real-time continuous monitoring of CTCs reveals that they occur at highly variable rates in a detection point over a period of time (e.g., a range of 0–54 CTCs per 5 min). These same fluctuations in CTC numbers were observed *in vivo* in epithelial and non-epithelial metastatic tumors, in different stages of tumor progression, and in different vessels. These temporal CTC fluctuations can explain false negative results of a one-time snapshot test in humans. Indeed, we observed wide variations in the number of CTCs in subsequent blood samples taken from the same metastatic melanoma patient, with some samples being CTC-free. If these phenomena are confirmed in our ongoing *in vivo* clinical trials, this could support a personalized strategy of CTC monitoring for cancer patients.

## 1. Introduction

Circulating tumor cells (CTCs) are unique biomarkers of deadly metastasis and the main participants in all steps of metastatic progression (metastatic cascade), including intravasation of CTCs into circulation, their dissemination via body fluids (e.g., blood lymph, cerebrospinal fluid), arrest in the capillaries, extravasation and entrance into target organs, and colonization [1]. Such obvious significance has stimulated intensive investigations of CTCs during the last decade [2,3,4,5,6,7,8,9,10,11]. Today, there are ≈400 registered clinical studies investigating CTCs [12]. It has been discovered that CTCs hold potential as prognostic biomarkers for therapy and survival [2,4,5,6,8,12,13,14,15,16,17,18]. Nevertheless, Budd *et al*. [19] reported that CTC numbers do not always correlate with the status of primary tumors and metastases obtained by computed tomography and magnetic resonance imaging [19]. Furthermore, it was shown that 36% of patients with metastatic breast cancer had undetectable CTC status [20]. This discordance could be caused by the fluctuations in the rate of CTC release into circulation. Indeed, the structural and functional heterogeneity of primary tumors and host environments as well as their changes over a disease progression suggest that the number of CTCs can vary from individual to individual and at different time points. This has been shown in blood samples *in vitro* taken from the same mouse where different numbers of CTCs were found in these samples [21]. However, dynamic features of CTCs are not fully explored by *in vitro*/*ex vivo* approaches allowing only a snapshot of CTCs’ behavior and does not provide long-term, real-time data about their dynamics. Such biological and technical challenges limit the clinical utility of CTCs as a biomarker of metastasis and, eventually, prevent improving diagnosis of the metastatic process in patients [20,22,23,24,25].

In the last decade, the technical problem was solved using *in vivo* flow cytometry (FC) with preferentially photoacoustic (PA), photothermal, and fluorescent detection schematics [9,10,11,14,15,26,27,28,29,30,31]. Our and others’ numerous preclinical studies of metastatic tumors (e.g., melanoma, breast cancer) have successfully shown that *in vivo* continuous monitoring of CTCs in circulating blood, lymph and cerebrospinal fluid have an unprecedented sensitivity and a high specificity, compared to *ex vivo*/*in vitro* CTC assays. To detect bulk CTCs and their subpopulations, such as cancer stem cells, a wide range of contrast agents have been used, including intrinsic chromophores (e.g., melanin), genetically encoded fluorescent proteins (e.g., green fluorescent protein [GFP]), and bioconjugated fluorescent dyes, quantum dots or nanoparticles (e.g., gold nanorods). Among different FC schematics, PA flow cytometry (PAFC) is the most clinically relevant method because PAFC (1) operates with laser energies parameters that are safe for humans; (2) uses nontoxic (e.g., intrinsic natural absorbers, such as melanin to track melanoma CTCs) or low toxic (e.g., magnetic and gold nanoparticles) contrast agents; and (3) provides higher sensitivity and resolution in deeper tissue (up to a few cm) compared to other optical modalities [11,28]. In the study described here, we use PAFC to define individual patterns and features of CTC dynamics during primary tumor growth and metastasis development.

## 2. Results and Discussion

### 2.1. Control Measurements on Healthy Mice

The PAFC was calibrated using mouse ear and skin blood vessels (50–70 µm and 150–250 µm in diameter, respectively) without any interventions. We did not observe PA signals above the blood background at laser wavelength of 1,064 nm. For GFP detection, we used fluorescence flow cytometry (FFC) with a continuous wave (CW) laser (wavelength, 488-nm, power, 2 mW) and determined signal-amplitude threshold in each channel as the mean and a multiple of the standard deviation (typically 5 SDs) of the autofluorescence background signals. Signals having higher amplitude than this threshold were associated with CTCs.

### 2.2. A Primary Tumor Size Is Not a Strong Indicator of CTC Quantity

**Figure 1:**
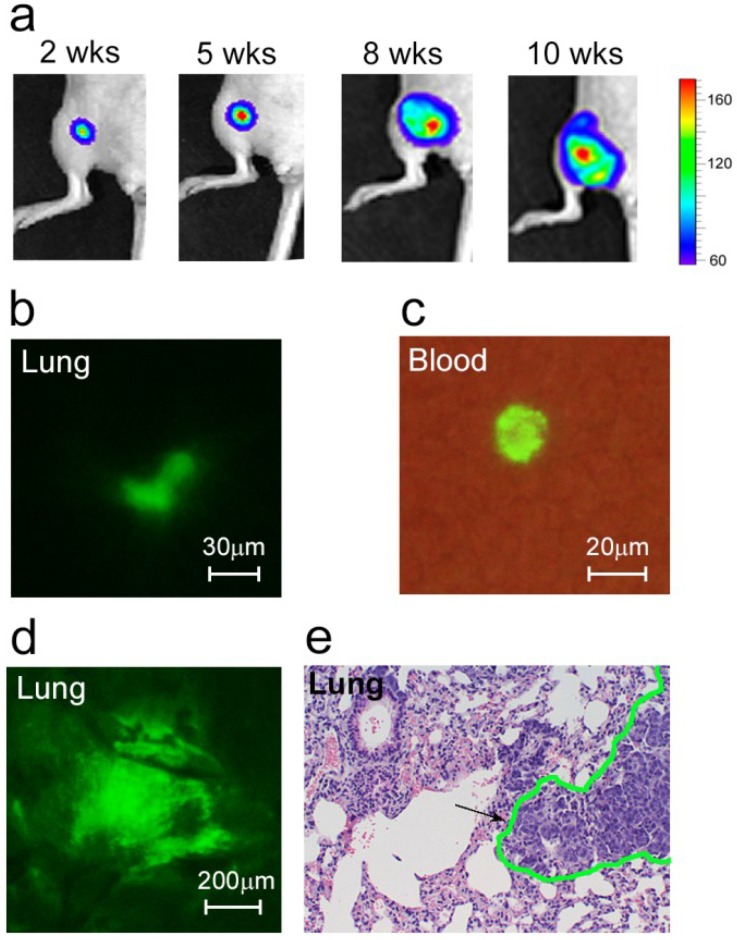
Development of metastatic breast cancer. (**a**) Growth of primary tumor; (**b**) Colony of cancer cells (green) in the lung at week 2 after tumor inoculation; (**c**) A CTC (green) in the blood sample obtained from a mouse at week 1 after tumor inoculation; (**d**,**e**) Lung metastases at week 8 after tumor inoculation confirmed by two independent methods: fluorescence image of lung *ex vivo* (**d**) and histological staining (H&E) of a lung section (**e**). Black arrow and green line in (**e**) show metastasis.

We inoculated mice with breast cancer in the mammary glands, which caused orthotopic primary tumor growth (Figure 1a) and metastatic disease development (Figure 1b,d,e). Specifically, we used MDA-MB-231 cells expressing GFP and luciferase (MDA-MB-231-luc2-GFP). With GFP, we were able to detect most of the bulk CTCs in blood circulation using FFC, and were able to verify these *in vivo* results by fluorescent imaging blood samples. Luciferase was used as a well-known advanced marker to identify metastasis by whole-body imaging.

As expected, the earliest micrometastases (week 2 after inoculation) were imaged in the sentinel lymph node (SLN) as the first metastatic site. These metastatic lesions affected 20% of the mice. At this time, a few colonies of tumor cells were also imaged in the lungs (Figure 1b). In week 5–6, the metastatic disease had progressed, characterized by the development of distant micrometastases (mainly in the lungs) in approximately half of the animals. In week 8–10, micro- and macrometastases (e.g., lungs, lymph nodes, liver, bones) were detected in 88.9% of the tumor-bearing mice (Figure 1d,e).

**Figure 2:**
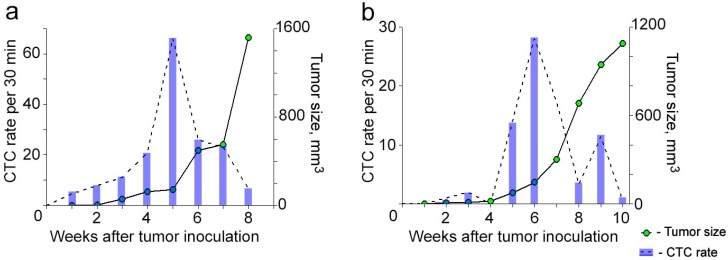
Correlation of CTC kinetics with tumor growth in breast cancer based on FFC data and whole body imaging, respectively. (**a**) Individualized CTC dynamics maximize before the primary tumor starts progressively growing; (**b**) Individualized CTC dynamics with two peaks. Tumor volume was calculated by formula: ½ × long diameter × short diameter^2^ [32].

To explore the individual dynamic of CTCs during primary tumor progression and metastases, we periodically applied FFC to each animal. In the majority (>90%) of mice, the signals associated with bulk CTCs appeared from week 1 of tumor development (Figure 1c and Figure 2). Monitoring CTCs afterwards revealed interesting facts. In most animals (83%), the highest CTC rate was detected when the primary tumor was small and slowly growing (Figure 2a). Later, when the primary tumor started progressively growing, the CTC rate decreased. The occurrence of this phenomenon varied from mouse to mouse likely due to specific features depending on host environment. In a few cases when the tumor was large, we observed a second increase in CTC number, but this CTC peak was smaller than the earlier one (Figure 2b).

### 2.3. Fluctuating Dynamics of Spontaneous CTCs in Breast Cancer

**Figure 3:**
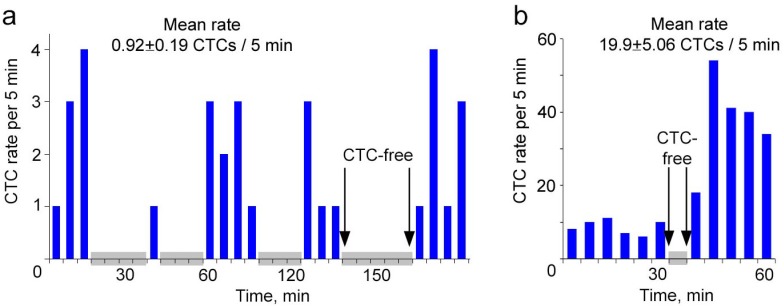
Real-time monitoring of spontaneous CTCs in ear blood vessels of tumor-bearing mice. (**a**,**b**) CTC dynamics during continuous FFC without averaging in mice at week 2 (**a**) and 5 (**b**) post inoculation. Grey squares show time-intervals of CTCs absence in a detection area.

Continuous real-time monitoring of CTCs using FFC and calculating their rate during the subsequent same time intervals revealed significant fluctuations in CTC count from interval to interval (Figure 3). When CTCs in circulation were relatively rare, their rate assessed every 5 min varied between 0 and 4 (Figure 3a). CTC absence in the detected blood volume lasted up to 25 min periods. Notably, the mean value of CTC rate per 5 min masked these interesting facts. For the same experiment, if we divided the total number of CTCs, which we counted for 60 min, by 12, the rate was 0.92 ± 0.19 CTCs/5 min. The intermitted and fluctuated dynamics of CTC rate in circulation was also observed if the CTC number increased. At the higher mean level of 19.9 ± 5.06 CTCs per 5 min, the number of CTCs assessed every 5 min ranged from 0 to 54 cells (Figure 3b). Taking into account that the average diameter of an examined ear vessels was 50 µm and the blood flow velocity was 3 mm/s [11], the CTC-free volume of blood was 0.45% of the total blood volume (~2 mL for a mouse) if the CTC count was relatively low (0.92 ± 0.19 CTCs/5 min), and 0.09% if CTC count increased by almost 20 times (19.9 ± 5.06 CTCs/5 min).

### 2.4. Dynamics of CTCs in Mice with a Primary Tumor Growing from Breast Cancer Stem Cell

**Figure 4:**
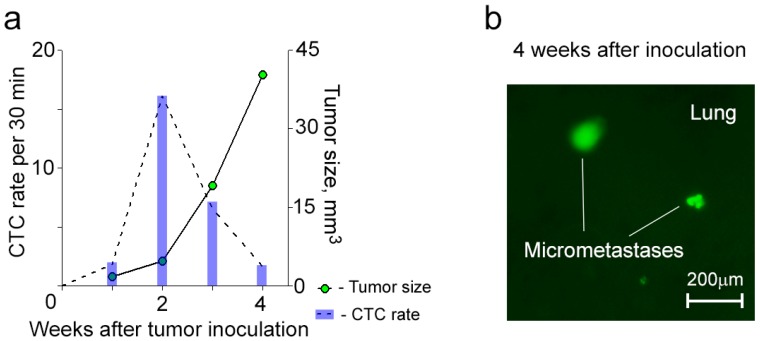
Dynamics of CTCs in mice after inoculation of CSCs. (**a**) A typical example of the dynamics of CTCs with their maximum number before the primary tumor starts growing progressively; (**b**) Lung micrometastases (green).

To determine whether an aggressive primary tumor will release CTCs with aforementioned phenomena, we used conventional flow cytometry to sort MDA-MB-231-luc2-GFP cells for CD44+/EpCam+/CD24− that characterized tumor-initiating cancer stem cells (CSCs) [31,32]. Sorted cells (*i.e.*, CSCs) were inoculated into a mammary gland of the first group of mice, and non-sorted cells (*i.e.*, bulk tumor cells) were inoculated into a mammary gland of the second group of mice. Although the amount of inoculated CSCs (1.7 × 10^5^ cells per mouse) was almost 30 times less than the amount of inoculated bulk tumor cells (5 × 10^6^ cells per a mouse), all mice from the first group developed metastatic disease (e.g., lung, liver) within 4 weeks after inoculation (Figure 4). In this group, CTCs were detected in circulation starting from week 1 after inoculation, and their dynamic was characterized by the maximum CTC number at an early stage of disease and their gradual decrease during primary tumor growth and metastasis progression (Figure 4a). In addition, mice after inoculation of CSCs exhibited similar, fluctuated dynamics during FC procedure like those obtained for CTCs after inoculation of bulk tumor cells.

### 2.5. Fluctuated CTC Dynamics in a Non-Epithelial Metastatic Tumor

**Figure 5:**
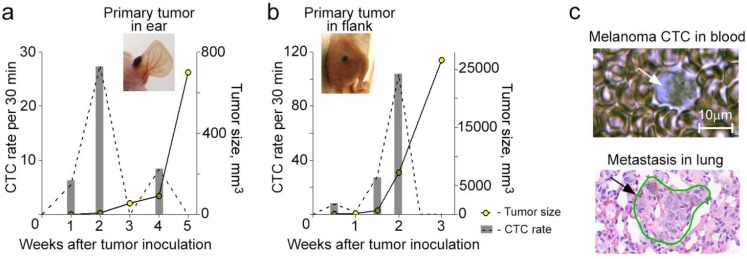
Dynamic interrelationship of CTC count and primary tumor size in the progression of metastatic melanoma. (**a**,**b**) CTC dynamic with the maximum number before the primary tumor starts progressively growing in the mice with a primary tumor in ear (**a**) and flank (**b**); (**c**) Images of a melanoma CTC in a fresh blood sample *ex vivo* (top) and a lung metastasis in a histological section *in vitro* (bottom) obtained at week 4 post tumor inoculation. White arrow (top image) show the pigmented CTC; black arrow and green line (bottom image) indicate metastasis.

Skin melanoma, as a highly metastatic mesenchymal tumor, was developed through inoculating melanoma cells (B16F10 cell line) into mouse ear (Figure 5a, insert) or flank (Figure 5b, insert) and was characterized by early production of CTCs (Figure 5a–c, top) and quick development of metastatic disease (Figure 5c, bottom). The CTCs were periodically monitored by PAFC over the disease course. The detection was label-free and based on the expression of high-absorbing melanin in melanoma CTCs. The experimental design was the same as described above for breast carcinoma. First, CTC number was calculated by averaging the results from groups of mice and, as expected, positively correlated with the size of the primary tumor, which were in line with the data previously reported by us [11,15]. However, the assessment of individual CTC dynamic for each mouse revealed that CTC rate in some cases did not correlate with the size of primary tumor. Around 35% of mice exhibited a significant increase in CTC number before intensive primary tumor growth (Figure 5a,b). These results indicate that the melanoma, like breast carcinoma, can shed high amounts of CTCs at a stage where the primary tumor is relatively small.

Furthermore, continuous PAFC monitoring of ear vessels during each procedure showed that melanoma CTCs, similar to the breast cancer CTCs, have the fluctuated dynamic and intermitted appearance in a detection area.

**Figure 6:**
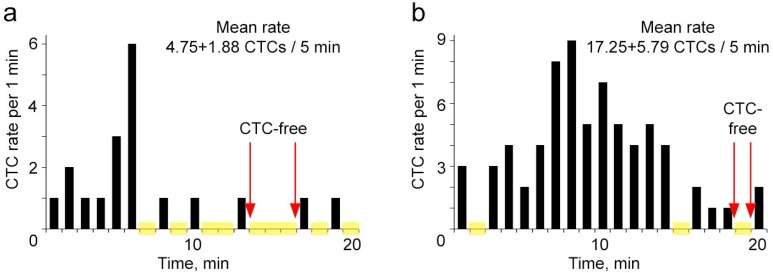
Real-time continuous PAFC monitoring of spontaneous CTCs in ≈200 µm blood vessels of mice with the primary melanoma tumor in the flank. (**a**) CTC number at week 1 after inoculation; (**b**) CTC number at week 2 after inoculation. Yellow squares show time intervals of CTC absence in a detection area. Laser parameters: 1,064 nm, 0.5 J/cm^2^, 10 kHz.

Next, we examined whether the fluctuation of CTC rate is an attribute of only small ear vessels or if it is kept in relatively large vessels. For this, PAFC was applied to skin vessels that had 3–5-times larger diameter (150–250 µm) and higher blood flow velocity than ear vessels. Monitoring the skin vessels at different stages of tumor development also showed that CTCs were absent for up to 3 min in the detection volume (Figure 6). This means that ≈1.4% of total blood volume may be CTC free for the typical mouse skin vessel with a diameter of 200 µm and a blood flow velocity of 5 mm/s.

### 2.6. Detection of CTCs in Blood Samples Subsequently Taken from Patients with Metastatic Melanoma

Nine milliliters of blood was drawn subsequently in three different tubes (3 mL per tube) using the same syringe from patients with stage IV melanoma. Thus, we were able to examine three equal volumes of blood subsequently flowing through an injection point. The CTCs were counted with conventional magnetic-activated cell sorting (MACS) assay. Averaged data obtained from three samples showed the mean CTC number per 1 mL was 0.3; 1; 1; 2.6; 2.3; 1.3; and 10.6 for patient 1 to 7, correspondingly. However individual analyzing each of three samples from the same patient showed different amounts of CTCs (Table 1). In some cases, CTCs were found in only one of three samples. This indicates that 0.12% of the patient blood volume was CTC free. Taken together, our clinical results indirectly confirm the fluctuated and intermitted dynamic of CTCs in circulation.

**Table 1:** CTC number per 1 mL of blood obtained from seven melanoma patients.

	1st sample	2nd sample	3rd sample
Patient #1	0	0	1
Patient #2	0	0	3
Patient #3	1	0	2
Patient #4	1	3	4
Patient #5	1	4	2
Patient #6	0	2	2
Patient #7	8	11	13

### 2.7. Discussion

Despite substantial efforts, understanding behaviors of CTCs and mechanisms of their dissemination are not completely clear [20,21,22,23,24,25]. As a result, we currently do not have the clinical capability to intervene and stop CTC dissemination, and therefore the metastatic process in patients [16,17,18]. One of the main biological challenges is heterogeneity of the CTC population. The significance of many molecularly different CTC subpopulations (*i.e.*, cancer stem CTCs and CTCs with epithelial to mesenchymal transition [EMT]) has been clarified in the last decade [3,7,31,33,34], but quantitative and temporal heterogeneity of CTCs remains poorly understood. This, at least partly, can be explained by technical limitations of existing CTC assays that mainly use *in vitro* testing of blood samples. Indeed, it is difficult to explore dynamic behavior of CTCs with *in vitro* approaches because they do not allow continuous monitoring of CTCs, and they have relatively low sensitivity, which is limited by the volume of a blood sample (typically 1–10 mL for humans). This problem can be solved with *in vivo* FC [26,28], which allows real-time (*i.e.*, continuous) monitoring of CTCs over a period of time in almost the whole blood volume (≈1–3 L for humans) that can significantly increase the sensitivity (up to 10^2^ fold) compared to *in vitro* assays.

In our experiments using *in vivo* FC, we found that, in some cases, the maximum amount of CTCs appeared in circulation in the early stage of tumor development. Specifically, we detected CTCs in real-time *in vivo* in mice with orthotopic xenograft breast cancer and skin metastatic melanoma. Both epithelial (breast cancer) and non-epithelial (melanoma) tumor models revealed CTCs’ early appearance (starting from week 1 of tumor development), which can be related to the model of parallel progression [35] of the primary tumor and metastasis and can support a recent mathematical model predicting the release of CTCs by very small primary tumors [18]. In the preclinical models of metastatic tumors, we found that not all mice exhibited a traditional relationship of increasing CTC number with primary tumor growth. In the most animals (83%) with orthotopic breast cancer and in 35% with melanoma, the highest level of CTCs was detected at the early stage of disease when the primary tumor was small and grew relatively slow. Later, when the tumor started growing progressively, the number of CTCs decreased 2–10 times. In some animals with both types of tumor, there was a second peak of increasing CTCs at the advanced stage of disease, but this late peak was smaller than the early one. Thus, the existence of this phenomenon in two different tumors allows us to suggest its general character for metastatic disease. The difference in its expressiveness from mouse to mouse as well as for breast cancer and melanoma is likely explained by specific features depending on type of tumor and host environment. Furthermore, our findings demonstrate that such interesting CTC dynamics are attributed to aggressive primary tumors growing from CSCs.

The particular mechanisms of these phenomena are not currently clear. They can be related to high primary tumor heterogeneities, various intratumor vascularity and blood flow dynamics, vessel damage in peripheral zones of a primary tumor, release of CTCs from growing metastases, temporal and individual changes in a balance between intavasation and extravasation rates of CTCs as well as varying of average CTC lifespan in circulation [1,7,9,11,14,28,29,36,37]. We believe that future studies using *in vivo* FC and other advanced technologies could elucidate the mechanisms behind CTC fluctuations.

The open question is whether these dynamics are reproducible in humans. We hope that our upcoming clinical trials using *in vivo* PAFC will provide the answer on this question. If the existence of these phenomena is confirmed, it might be used to explain the presence of multiple metastases in some patients at the time of initial diagnosis [1] and will support a personalized strategy for clinical CTC monitoring.

We also discovered that CTCs are not uniformly distributed in circulating blood, and their amount in a detected volume (or withdrawable volume) is not consistent over a period of time. Our *in vivo* continuous monitoring the circulating blood of mice with metastatic breast cancer and melanoma demonstrates CTCs’ highly fluctuating behavior and their intermitted appearance in the detection area. CTC fluctuations were observed at different stages of tumor progression and in various vessels. Irregular temporal appearance of CTCs in a detection point could be the result of the fluctuating process of shedding cells from the primary tumor or/and arresting of CTCs within the microcirculatory network followed by releasing several CTCs together.

While the specific mechanisms of dynamic fluctuations of CTC rate are not quite clear, we did find indirect evidences of these fluctuations in blood samples from patients with metastatic melanoma. Three blood samples of the same volume were subsequently taken over a period of time and exhibited high sample-to-sample variability in the number of CTCs. Some samples were CTC free, representing an example of false negative results. Specifically, we showed that up to 6 mL of blood (≈0.12% of a total blood volume of ≈5 L) taken from some patients with advanced melanoma were CTC free. These findings can be explained by the aforementioned temporal fluctuations of CTC number.

From a clinical point of view, thus, our results suggest that one of the obstacles of *in vitro*/*ex vivo* methods is that a blood sample allows only a snapshot of CTCs in the blood at a specific time point, without long-term, real-time monitoring. Future studies could be aimed at providing personalized real-time CTC monitoring in patients by developing *in vivo* clinical prototypes of PAFC that are highly sensitive and capable of assessing CTCs in real-time and in larger blood volumes circulating through peripheral vessels.

## 3. Experimental

### 3.1. Cells

Human breast cancer cells (MDA-MB-231-luc2-GFP Bioware^®^ Ultra Green cell line, Caliper Life Science, Hopkinton, MA, USA) and mouse melanoma cells (B16F10, American Type Culture Collection, Manassas, VA, USA) were cultured according to the vendors’ specifications. Viable cells were resuspended in phosphate buffered saline (PBS) or in a sample of mouse blood. To collect breast CSCs, the bulk MDA-MB-231-luc2-GFP cells were sorted with conventional flow cytometry for CD44+/EpCam+/CD24−.

### 3.2. Animal Models

Animals were used in accordance with a protocol approved by the University of Arkansas for Medical Sciences Institutional Animal Care and Use Committee. Nude mice (nu/nu), 8–10 weeks old, weighing 20–30 g, were procured from a commercial source for use in the experiments. The animals were anesthetized by isoflurane and placed on a heated microscope stage (at 38 °C [body temperature]). To observe abdominal-skin blood vessels, a fold of abdominal skin was placed between two moistened glass coverslips.

We used the xenograft orthotopic mouse model of breast cancer and two mouse models of metastatic melanoma. To establish primary tumors, tumor cells were inoculated using 1-mL syringe with a 30-gauge needle: 5 × 10^6^ bulk MDA-MB-231-luc2-GFP breast cancer cells in 5 µL of PBS were injected into a mammary gland; 1.7 × 10^5^ breast cancer stem cells in 5 µL of PBS were injected into a mammary gland; and 1 × 10^6^ B16F10 mouse melanoma cells in 5 µL of PBS were injected either into the ear or into the skin of flank. The mice were examined weekly with FFC or PAFC *in vivo* for 3–4 weeks for melanoma and 8–10 weeks for breast cancer.

The breast CTC rate was measured with FFC in ear blood vessels with a diameter of 50–70 µm and blood velocities of 1–3 mm/s. The growth of the primary tumor and metastases was imaged by an IVIS Spectrum imaging system (Caliper Life Science, Hopkinton, MA, USA).

The melanoma CTCs were counted with PAFC in the ear blood vessels with similar size as well as in skin blood vessels of the abdominal wall with a diameter of 150–250 µm and blood velocities of 3–5 mm/s.

Control measurements were performed on intact vessels of five healthy mice. For valid comparison of the data, both FCs were performed with the same parameters (laser power, monitoring time, *etc*.) on vessels of similar diameters to those in the experimental groups. In addition, tissue samples from healthy mice were used to estimate autofluorescence level in microscope images.

### 3.3. Patient Samples

Blood samples were obtained from seven melanoma patients at stage IV during autumn 2013 with informed consent according to the IRB-approved protocols (133965). A total blood volume of 9 mL was collected from each patient in three *EDTA tubes* (BD, Franklin Lakes, NJ, USA) for *ex vivo* CTC enumeration. A total of 3 mL patients’ blood was processed for one test using magnetic-activated cell sorting (MACS) with the protocol provided by the vendor. Specifically, the CTCs in the examined samples were detected using MCSP–MNP conjugates. First, to isolate mononuclear cells (MNCs), each whole blood sample was diluted 1:1 with PBS and carefully loaded onto Ficoll–Paque solution (1.077 g/mL, Sigma-Aldrich Co., St. Louis, MO, USA). After density gradient centrifugation at 400 × *g* for 30 min at room temperature, MNCs were removed from the interphase, washed twice with PBS, mixed with Anti-Melanoma (MCSP) MicroBeads and incubated for 30 min at 4 °C. Cells isolated from MACS (Miltenyi Biotec INC., Auburn, CA, USA) were prepared for immunocytochemical staining using melanoma cocktail primary antibodies (HMB-45+MART-1+Tyrosinase), HiDef-Detection^TM^ HRP polymer system, and DAB kit (Cell Marque Corporation, Rocklin, CA, USA).

### 3.4. *In Vivo* Flow Cytometry (FC)

Detection of CTCs with FC *in vivo* included noninvasive irradiation of a vessel with low laser energy fluence in the range of 10–50 mJ/cm^2^ (*i.e.*, within the laser safety standard). Lasers were delivered with a microscope schematic as previously reported [11,15,21,22,24]. Specifically, FFC was used to detect breast CTCs that were expressing GFP with an excitation wavelength of 488 nm and an emission wavelength of 509 nm. PAFC at 1,064 nm was used for label-free detection of melanoma CTCs, which have intrinsically high-absorbing melanin and serve as a strong PA biomarker.

The setup incorporating PA and fluorescent modules was based on a customized Nikon Eclipse E400 microscope platform (Nikon Instruments Inc., Melville, NY, USA) that was converted into an invert system [24]. The setup was equipped with two lasers: pulsed laser for PA detection and CW laser for fluorescence excitation. High pulse rate Yb-doped fiber laser operated at 1,064 nm with a maximum energy of 30 µJ, 10 ns pulse width at a pulse repetition rate of 10 kHz (MOPA-M-10, MultiWave Photonics S.A., Maia, Portugal). A CW diode 488 nm laser (IQ1C45 (488-60) G26, Power Tech., Alexander, AR, USA) with 2 mW in the sample was used for fluorescence excitation. Two dichroic mirrors were used to combine pulsed and CW lasers. Laser beams were focused into mouse vessel by a 40× microobjective (Plan Fluor, NA 0.75; Nikon Instruments, Inc.). In FFC, these dichroic mirrors and microobjective were also used to collect fluorescence from CTCs genetically encoded with GFP. Additionally, in FFC, the emission bandpass filter with spectral band centered at 520 nm and a bandwidth of 15 nm was used (Semrock, Inc., Rochester, NY, USA). A variable-width slit in the front of the photomultiplier tube ([PMT] R928, Hamamatsu, Co., Bridgewater, NJ, USA) was used to control axial resolution to provide detection of CTCs in a whole vessel, while efficiently filtering out-of-plane autofluorescence of the tissues.

To detect laser induced PA waves from CTCs in PAFC, the light-weight PA transducer (V312-SU, 12 mm focal distance, Panametrics NTD Inc., Waltham, MA, USA) was gently placed on the surface of the skin close to the investigated vessel. Warm water (or gel) was applied for the acoustic coupling between transducer and tissues.

Cylindrical lenses (*f* = 250 mm) provided linear configuration (e.g., 10 × 80 μm for ear vessels) for both pulsed and CW laser beams covering the whole vessel diameter, which allowed detection of all CTCs passing through the vessel cross-section.

Both PAFC and FFC systems were controlled using a workstation (Precision 690, Dell Inc., Round Rock, TX, USA) and custom software (LabView, 8.5, National Instruments, Austin, TX, USA). In FFC, signals after PMT were continuously sampled at 4 MHz by a high-speed digitizer (PCI-5124, National Instruments) and downsampled to 10 kHz by averaging 400 points. In PAFC, ultrasound transducer signals were digitized for 4 µs after each laser pulse by the same data acquisition card. For each signal, the software determined peak-to-peak signal amplitude, which was further referred as PA signal. Fluorescence and PA signals were then combined into traces, displayed in real-time, and saved for later processing. Traces were analyzed to identify peaks over selected threshold and measure location, amplitude and width of each peak.

For each acoustic signal, the software determined peak-to-peak signal amplitude, which was further used as PA signal. Fluorescence and PA signals were then combined into traces, displayed in real-time, and saved for later processing. Traces were analyzed to identify peaks over selected threshold and measure location, amplitude and width of each peak.

### 3.5. High-Resolution Fluorescent Imaging

For imaging tissue samples or CTCs in blood samples *ex vivo*/*in vitro*, we used an Olympus IX81 inverted microscope that was equipped with a cooled color charge-couple device (CCD) camera (DP72, Olympus) and a black-and-white highly sensitive CCD camera (Cascade:512; Photometrics/Roper Scientific, Inc., Tucson, AZ, USA). Images were obtained in either transmission mode or fluorescence mode with an emission filter of 530 ± 30 nm. Images were acquired, combined (if necessary) and processed in *Adobe Photoshop 7.0.1* software (Adobe Systems, San Jose, CA, USA) and *Image J 1.46* for Windows.

### 3.6. Terminal Blood Collection and Tissue Procurement for Histological Tests and *ex Vivo* Imaging

At the end of *in vivo* measurements, blood was collected through a cardiac puncture with a plastic sterile syringe containing anticoagulant to prepare *ex vivo* blood samples. After blood collection, animals were euthanized and tissues were taken for *ex vivo*/*in vitro* tests. Liver, lymph nodes, lungs, bones and brain were extracted for *ex vivo* transmission and fluorescent (530/30 nm filter for GFP) optical imaging with different magnifications (objective lenses of 20× and 40×). Multiple organs were also fixed in phosphate-buffered 10% formalin (pH 7.2) and then embedded in paraffin for histological examination with hematoxylin and eosin (H&E) staining.

### 3.7. Statistical Analysis

MATLAB 7.0.1 (The MathWorks, Inc., Natick, MA, USA) was used for statistical analyses. Results were expressed as means ± SEM of at least three independent experiments. Probability of *p* < 0.05 indicated a significant difference.

## 4. Conclusions

In our study, we found that the number of CTCs for selected preclinical models does not always correlate with the primary tumor size. The highest CTC number may appear in circulation at the early stage of disease before the primary tumor starts growing progressively, and CTC number may significantly decrease when then tumor is large. This phenomenon of CTC dissemination has general character with specific features depending on the type of tumor and individualized disease development. Furthermore, real-time continuous monitoring of spontaneous CTCs showed varying rates of CTCs in a detection area. Fluctuations in CTC numbers were seen in epithelial and non-epithelial metastatic tumors, in animals and humans, in different stages of tumor progression and in various vessels. Our findings hold promise to provide new insights on mechanisms of metastatic diseases and may have diagnostic implication towards developing advanced personalized and early diagnosis, allowing for well-timed therapy that is more effective. Further studies with comprehensive statistical analysis and trials in humans will provide the insight on these phenomena.
